# Electronic Properties of a Synthetic Single-Crystal Diamond Exposed to High Temperature and High Radiation

**DOI:** 10.3390/ma13112473

**Published:** 2020-05-29

**Authors:** Andreo Crnjac, Natko Skukan, Georgios Provatas, Mauricio Rodriguez-Ramos, Michal Pomorski, Milko Jakšić

**Affiliations:** 1 Division of Experimental Physics, Ruđer Bošković Institute, 10000 Zagreb, Croatia; Natko.Skukan@irb.hr (N.S.); Georgios.Provatas@irb.hr (G.P.); Mauricio.Rodriguez@irb.hr (M.R.-R.); Milko.Jaksic@irb.hr (M.J.); 2 CEA-LIST, Diamond Sensors Laboratory, F91191 Gif-sur-Yvette, France; michal.pomorski@cea.fr

**Keywords:** diamond, detector, high-temperature, ion beam, radiation damage

## Abstract

Diamond, as a wide band-gap semiconductor material, has the potential to be exploited under a wide range of extreme operating conditions, including those used for radiation detectors. The radiation tolerance of a single-crystal chemical vapor deposition (scCVD) diamond detector was therefore investigated while heating the device to elevated temperatures. In this way, operation under both high-temperature and high-radiation conditions could be tested simultaneously. To selectively introduce damage in small areas of the detector material, a 5 MeV scanning proton microbeam was used as damaging radiation. The charge collection efficiency (CCE) in the damaged areas was monitored using 2 MeV protons and the ion beam induced charge (IBIC) technique, indicating that the CCE decreases with increasing temperature. This decreasing trend saturates in the temperature range of approximately 660 K, after which CCE recovery is observed. These results suggest that the radiation hardness of diamond detectors deteriorates at elevated temperatures, despite the annealing effects that are also observed. It should be noted that the diamond detector investigated herein retained its very good spectroscopic properties even at an operation temperature of 725 K (≈2% for 2 MeV protons).

## 1. Introduction

It is expected that diamond, as a large band-gap semiconductor, is able to maintain good electronic properties at working temperatures much higher than those applicable for silicon. One can calculate that the intrinsic carrier concentration in diamond at 1000 K is lower than that for silicon at room temperature [1], which indicates that diamond electronic devices should be able to operate at this and even higher temperatures. This fact makes diamond the material of choice for radiation detection at high temperatures, where the application of other semiconductor materials would be improbable. Although there are an extensive number of publications dealing with the radiation hardness of diamond detectors [2,3,4], there is limited information about detectors operating in both high-radiation and high-temperature environments. Concerning the upper limits of the diamond-based detector operating temperature, in recent years, several authors have shown that significant degradation of signal properties and charge collection efficiency may already appear at rather low temperatures ranging from 370K to 600K [5,6,7]. These inconclusive results demonstrate that the effects of temperature on detector operation need to be studied more thoroughly. Specifically, the thermal properties of metal-semiconductor contact for different metals, the thermal stability of the most important charge traps from electrically active impurities in a diamond crystal lattice, the high-temperature resistance of the electronic-processing chain in the immediate surroundings of the detector, and other effects, including the thermal dependences of radiation hardness and polarization phenomena, should be investigated.

In this work, we attempt to contribute to the understanding of the above-stated effects by using a radiation sensor prepared specifically for high-temperature operation. We have investigated the electronic properties and radiation tolerance of the detector in response to MeV proton beams while increasing the operating temperature. The covered temperature range spanned from room temperature to 725 K, thus increasing the upper temperature limit reported in literature, where the characterization of the spectroscopic properties of diamond-based detectors is successfully performed. Influence of temperature on the radiation hardness has clear relevance for applications of diamond detectors in high radiation environments, such as fusion reactors and accelerator facilities. Furthermore, general diamond electronic properties at high temperatures are also of interest for the development of power semiconductor devices (diodes, switching devices) [8].

## 2. Materials and Methods

**Figure 1:**
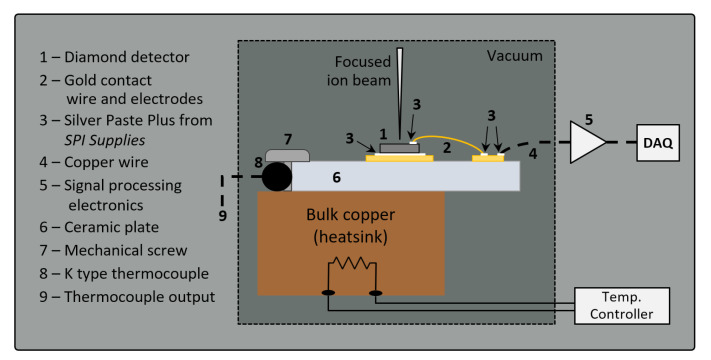
Schematic view of the setup in the ion microprobe chamber. The detector output signal was processed by the charge-sensitive preamplifier (ORTEC 142A) and the spectroscopy amplifier (ORTEC 570). Data acquisition was performed by the Canberra8075 ADC modules and SPECTOR software developed in-house [10]. For dark current-voltage characterization, a picoammeter with a voltage source was used (Keithley Instruments Inc. (Cleveland, OH, USA), model 6487).

The detector used in the experiment was prepared from an electronic grade single-crystal chemical vapor deposition (sc-CVD) diamond produced by Element Six Ltd. (Didcot, UK), with a nominal N concentration of <5 ppb and a <100> crystal orientation. To reduce the effects of polarization and to enable homogeneous depth distribution of defects when irradiated MeV energy proton beams, the diamond was thinned to a thickness of 65 µm by laser slicing and mechanical polishing conducted at Almax easyLab Inc. (Diksmuide, Belgium). Front and back electrodes with a 3×3 mm${}^{2}$ area were made by sputtering tungsten on the diamond surfaces. The thickness of the electrodes was approximately 200 nm. Tungsten was chosen with the intention that the metal-semiconductor contact should be able to withstand high temperatures without deterioration of electronic properties. Next, a metallized diamond sample was mounted (using high-temperature silver paste) on the ceramic plate with printed gold electrodes, as shown in the schematic drawing in Figure 1. The whole device was mounted on the universal sample holder for further tests in an irradiation chamber. The bias was applied through the front electrode, while the back electrode was grounded. The detector was heated by a resistive heater below the copper heat sink that was in contact with the back side of the ceramic plate. A type K thermocouple was used for temperature measurement. Heat distribution on the front side of the detector setup was tested using an infrared camera from Optris (Berlin, Germany), model PI640 [9], at different temperatures and showed an average deviation between the camera and the thermocouple reading of 5 K.

Room-temperature measurements showed no significant leakage current (I < 10 pA) up to ±63 V (0.97 V/µm). Irradiation and further spectroscopic characterization were performed in the ion microprobe chamber at the Ruđer Bošković accelerator facility [11]. Proton beams used in this experiment were focused to a spot size of ≈1 µm and could be scanned over the selected regions of the detector, in either low- or high-current mode. High-current mode (∼1–10 pA) was used for introduction of damage (irradiation), while low-current mode (<fA) was used for subsequent probing of the detector energy response with ion beam induced charge (IBIC) technique [12].

The IBIC technique used single ions to induce electron-hole pairs by ionizations that occur along the ion penetration trajectory. In the presence of an electric field, charge carriers move creating a charge signal at the sensing electrodes (IBIC signal). The signal is reduced if charge carriers (electrons or holes) are trapped by the electrically active defects in the crystal lattice. More information on the trapping-detrapping process and IBIC signal formation can be found in literature [13,14,15].

In the case of diamond, it is also important to minimize the density of injected charge in order to avoid a local buildup of the internal electric field due to the charge being trapped in deep traps. This internal field can screen the externally applied electric field (detector bias) and thus diminish the charge collection efficiency (CCE) in that area (polarization effect). Short range heavy ions produce high ionization in small volumes resulting in a higher probability for buildup of polarization [16,17,18]. In our experimental setting, long range protons were therefore used, producing no observable polarization in low-current mode.

It is important to note that the applied fluences during the IBIC probing cycles are insignificant and do not contribute to the defect production noticeable for the device operation.

Signal processing electronics for the IBIC technique are listed in the description of Figure 1. Energy calibration of the electronic chain was performed with a silicon surface barrier detector and a pulse generator. To calibrate the collected charge in the diamond detector, diamond e-h pair creation energy was assumed to be 13 eV [19], while a value of 3.62 eV was used for silicon.

## 3. Results and Discussion

To introduce damage into the diamond crystal lattice, a 5 MeV proton beam was used. Protons of this energy penetrate through the whole thickness of the sample. According to SRIM (Stopping and Range of Ions in Matter) Monte Carlo [20] simulations, the total deposited energy of these ions in 65 µm diamond is approximately 1.85 MeV. The defects were distributed almost homogenously throughout the whole depth of the sample, with an average of 0.12 vacancies produced per ion per micrometer of penetration. Unlike neutrons that produce mostly cluster defects and gamma radiation that produces dominantly point defects, protons induce both kinds of defects [21,22] and therefore were considered to be more appropriate for testing the radiation hardness of the detector. Only small portions of the detector area (100×100 µm${}^{2}$) were irradiated by the scanning proton microbeam, maintaining the overall detector electronic properties (leakage current) unchanged. In this way there was no need to irradiate many different detectors, which would induce uncertainties related to possible differences in diamond quality between the samples. All irradiations were performed in the same experimental settings, in two cycles. In each cycle, series of 3 detector regions were irradiated, namely, the Cycle 1 regions and Cycle 2 regions. Fluences that were deposited in these regions are listed in Table 1.

The deposited fluences were determined from the backscattered spectra collected from the gold-plated Al sheet chopper, positioned at the entrance of the microbeam chamber. The chopper was setup to periodically intercept the beam, in intervals of one second. Initially, to calibrate the chopper, the beam was terminated in the Faraday cup, where the accumulated charge was measured by the Ultra-sensitive charge digitizer, scaler and current indicator (Oxford Microbeams model OM35e). With this calibration procedure, the relation between the counts from the charge digitizer and the counts from the chopper was established. Consequently, the fluences during the irradiation steps were determined only from the chopper counts. The corresponding statistical uncertainty was obtained from the counts square root, which was better than 5%.

As seen from the data in Table 1, both the Cycle 1 and Cycle 2 irradiated regions have sections with low, medium and high deposited fluence. After irradiation of the Cycle 1 regions, the detector was heated to 725 K for approximately half an hour. Then, it was left to cool to room temperature, after which the Cycle 2 regions were irradiated. In this way, two sets of comparable irradiated fluences (spanning one order of magnitude) were created. The Cycle 1 set was first annealed by heating, while the Cycle 2 set was not. For probing and mapping of the changes in charge collection efficiency of the detector, a proton microbeam with a low current of 2 MeV was used. 2 MeV protons can be considered as a deep probe for our sample, since they are stopped in the bulk of the material with a penetration range of 24.5 µm (almost the half of the detector’s thickness). In this case, both electrons and holes contribute to the induced signal during the charge collection (but not equally), meaning that the results of this study do not completely distinguish e/h contributions in the signal response, as would be the case with a shallow probe. The applied electric field during all IBIC measurements was set to E = 0.092 V/µm. It is worth noting that this is a rather low field for typical operating conditions of diamond detectors [23,24]. In our case, this was the lowest field with which a full charge collection for 2 MeV protons could be achieved, and it was intentionally chosen to represent the worst-case scenario for radiation tolerance of the diamond. Furthermore, such conditions could reveal the effects of radiation damage by the highest sensitivity.

**Figure 2:**
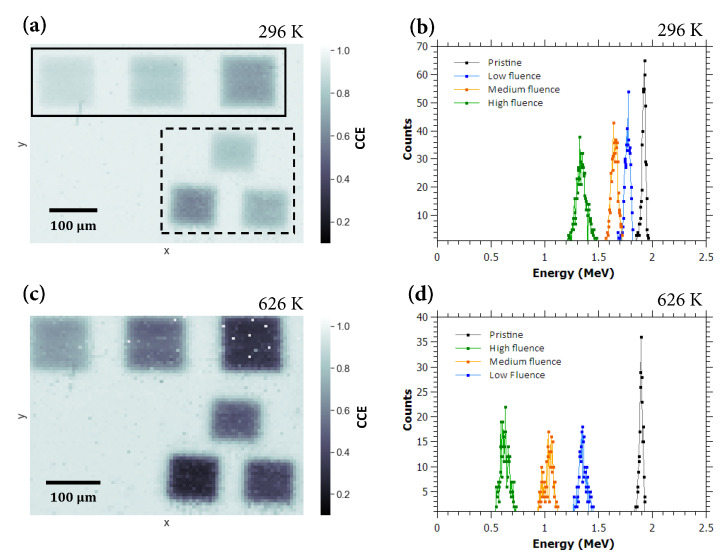
(**a**) IBIC map of the detector recorded at an operating temperature of 296 K. Cycle 1 regions are marked with a dashed line frame, and Cycle 2 regions are marked with a solid line frame. The applied electric field is 0.092 V/um; (**b**) IBIC spectra obtained in the Cycle 2 damaged regions and in the pristine diamond area, recorded at 296 K (Cycle 1 regions have been omitted for clarity of the plotted data); (**c**) IBIC map recorded at 626 K; (**d**) Same IBIC spectra as in (**b**), recorded at 626 K.

**Figure 3:**
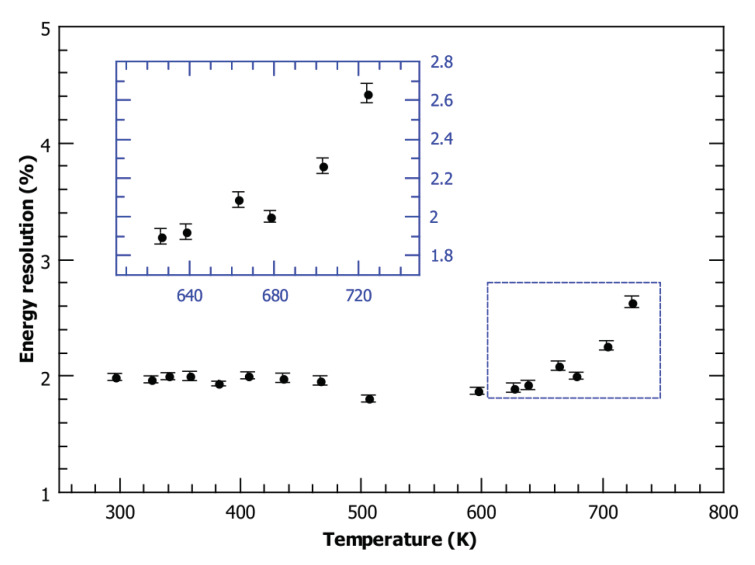
Temperature dependence of the energy resolution. The results were calculated from the peak width (full width at half maximum—FWHM) of the IBIC spectra recorded on the unirradiated detector area using 2 MeV protons as a probing beam.

Figure 2a shows an IBIC map of the detector area with clearly visible irradiated regions recorded at room temperature (296 K). We recorded an IBIC map on the same area for temperatures up to 725 K. Figure 2c displays the map recorded at 626 K. As shown in Figure 2b,d, the diamond detector that has been tested maintained good spectroscopic properties (energy resolution) even at elevated temperatures. Details of the detector performance in the pristine region are shown in Figure 3, where a plot of its energy resolution (FWHM), calculated from the Gaussian approximation of the IBIC spectra, is given for all tested temperatures. Although the pulse processing electronics were not aimed to be optimized for these measurements, it is evident that the energy resolution remains constant, at approximately 2% (40 keV FWHM), for temperatures up to 660 K. With further increases in the temperature, the resolution degrades somewhat to 2.64% for the highest measured temperature (725 K), which is still sufficient for most spectroscopic applications. These results demonstrate the advantage of diamond over other conventional semiconductors in radiation detection, as it retains its very good spectroscopic properties even at very high operating temperatures. Similar conclusions, but for lower operating temperatures, have been presented previously by other authors. In a recent work by P. Steinegger et al. [6], the α-spectroscopic resolution of a single-crystal CVD diamond detector was measured to be stable up to 453K, then progressive resolution worsening was observed in the range of 453–473K. Temperatures of approximately 470 K were reported to be the limit of the spectroscopic operation for diamonds tested with α sources by other authors as well: F. Nava et al. [25] using natural diamonds, and Tanimura et al. [26] using samples synthesized with the high pressure-high temperature (HPHT) method. However, Tsubota et al. [5] demonstrated that a sc-CVD diamond-based detector was able to collect spectra related to electron collection for conditions up to 573 K, while for the reverse polarity (hole collection), proper operation is stopped at lower temperatures. Similarly, Kumar et al. [27] tested several detectors based on single crystal diamonds using different materials for electrode deposition. One of the devices managed to perform energy spectroscopy without significant degradation of the energy resolution up to 573 K.

**Figure 4:**
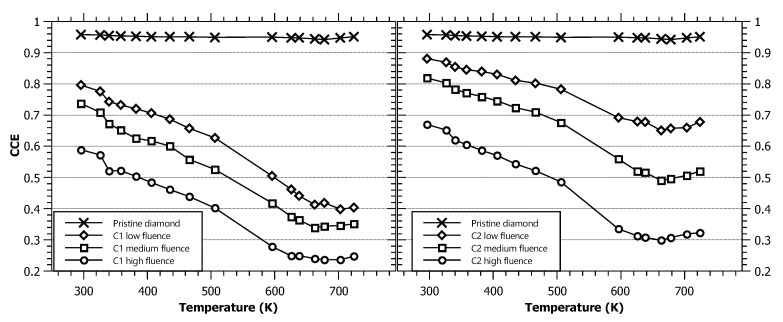
Temperature dependence of the CCE extracted from IBIC spectra obtained by 2 MeV protons for different detector target regions: six irradiated regions and the pristine diamond area. The solid lines serve as a visual guide. The results are grouped on the left panel for the Cycle 1 regions and the right panel for the Cycle 2 regions.

Another important spectroscopic property is the charge collection efficiency of the detector. We determined the CCE at elevated temperatures by simultaneous collection of energy spectra in pristine (unirradiated) and irradiated sample regions. This was already indicated in the IBIC maps displayed in Figure 2a,c, while the measurement results for all covered temperatures are plotted in Figure 4. The CCE in the pristine diamond regions at room temperature was measured to be 95.8%. This value remains practically constant with increasing temperature, with the lowest value measured being 94.2% at 678 K and increasing to 95.1% at 725 K, the highest temperature in the experiment. These results indicate that the full charge collection is maintained over the whole range of tested temperatures, and proper spectrometric performance is possible. Operation at even higher temperatures is to be evaluated in the future, with the upgraded construction of the mount for the heating setup, which would guarantee safe operation in the microprobe chamber with an increased thermal energy dissipation to the elements in direct contact with the heater stage.

Temperature seems to have a much larger effect on the charge collection process in irradiated regions. The left panel in Figure 4 lists the CCE values from the Cycle 1 irradiated regions, while the right panel is for the Cycle 2 regions. The CCE exhibits an overall decrease with increasing temperature for all irradiated regions, with the rate of decrease for the Cycle 1 regions being somewhat higher than that for the Cycle 2 regions. The CCE reached a minimum value for temperatures around 660 K. After this, saturation was attained. Additionally, it is noticeable that the CCE in Cycle 2 regions appears to start to recover at the end of the measured range. Recovery in the Cycle 1 regions (that were already pre-annealed at 725 K) seems to be less pronounced and even inconclusive. From the previous work of other authors [25,28], we know that the mobility of both electrons and holes in diamond decreases with temperature. Since the trapping time is inversely proportional to the carrier velocity [29], an increase in the time at which the charge carriers are captured by certain trapping centers will occur. This effect would explain the decreasing trend of the CCE due to charge carriers being trapped on defects induced during radiation. No changes were observed in the unirradiated diamond areas.

A potential explanation for the saturation of the collection efficiency at higher temperatures and even recovery at temperatures above 660 K is more ambiguous. Two effects probably contribute: thermally stimulated de-trapping and defect annealing. It has been observed that the thermal energy transferred to the charge carriers at temperatures close to 500 K enables their release from several deep traps typically present in CVD diamond [30]. As for the second effect, an annealing process starting at ∼670 K, related to the carbon interstitials, has been observed in HPHT-grown diamond samples [31]. Both of these effects can counter the decrease in collected charge in irradiated regions. Further experiments will be needed to determine whether the CCE behavior can be attributed to electron and/or hole transport properties and to explore the behavior of the CCE recovery at even higher temperatures.

**Figure 5:**
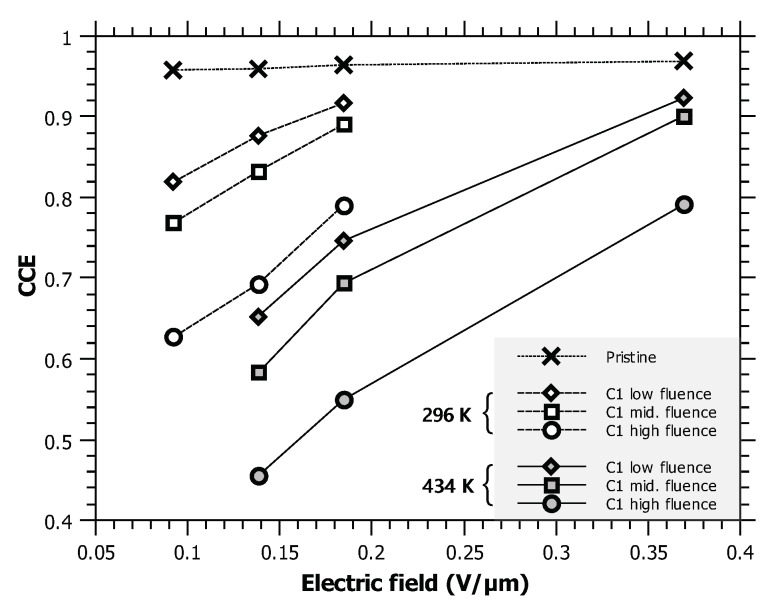
CCE vs. applied electric field for two temperatures: 296 K and 434 K. The field needed for the CCE of the lowest irradiated fluence to recover to 90% is only 0.185 V/µm at RT, and 0.37 V/µm at 434 K.

A possible way to mitigate the undesirable decrease in radiation hardness by increasing temperature would be the increase of the electric field. It has been demonstrated that the CCE of a sc-CVD diamond detector exposed to radiation damage can be significantly recovered if high electric fields (even up to 100 V/µm) are used [4]. As mentioned above, the electric field used in this measurement was very low (0.092 V/µm). To demonstrate the effect of increasing electric field, the CCE values extracted from the irradiated regions were recorded at room temperature (RT) and one elevated temperature (434 K). The results are displayed in Figure 5. The trend in the recovery of radiation tolerance is obvious; however, due to indications from other authors that the leakage current can increase dramatically for high voltage biasing at elevated temperatures [5,7,32], no further experiments in that direction have been performed.

## 4. Conclusions

In summary, a single-crystal CVD diamond detector, prepared specifically for high-temperature operation, has been tested at operating temperatures up to 725 K. The detector showed stable CCE (≈95%) and spectroscopic resolution (≈2%) for the full tested temperature range. The effects of temperature on the radiation hardness of the device operating at rather low electric fields (<1 V/µm) were much more pronounced, as the CCE showed a decrease of up to 40% after irradiation with 5 MeV proton fluences ranging from $10^{12}$ to $10^{13}$ cm${}^{-2}$. Saturation and recovery of the CCE is observed at temperatures higher than 660 K, which is very promising but requires further investigation. Analysis of the CCE trend at higher temperatures could provide further insights about competing contributions that affect CCE behavior. An upgrade to our heating setup is being prepared that will enable testing on higher temperatures as well as monitoring of the operating long-term stability at elevated temperatures. Moreover, further high temperature experiments are planned with short-range ion probes where electron and hole contributions in signal properties can be separated.

It seems that the critical components that limit successful application of diamond-based radiation sensors at high temperatures are not only inherent to the properties of the exploited diamond material, but also in the thermal resilience of the components that are used for device mounting and signal processing. More systematic studies that would address these issues are required to test the true operating limits of these devices.

## Figures and Tables

**Table 1:** Fluences and corresponding vacancy densities deposited during irradiation with 5 MeV protons.

	Low Fluence	Medium Fluence	High Fluence	
Fluence [cm${}^{-2}$]	$8.0\times 10^{12}$	$1.1\times 10^{13}$	$2.2\times 10^{13}$	Cycle 1 regions
Vacancy density [cm${}^{-3}$]	$6.2\times 10^{13}$	$8.6\times 10^{13}$	$1.7\times 10^{14}$	
Fluence [cm${}^{-2}$]	$3.8\times 10^{12}$	$8.3\times 10^{12}$	$1.6\times 10^{13}$	Cycle 2 regions
Vacancy density [cm${}^{-3}$]	$3.0\times 10^{13}$	$6.5\times 10^{13}$	$1.2\times 10^{14}$	

## References

[1] Sussmann R.S. (2009). CVD Diamond for Electronic Devices and Sensors.

[2] De Boer W., Bol J., Furgeri A., Müller S., Sander C., Berdermann E., Pomorski M., Huhtinen M. (2007). Radiation hardness of diamond and silicon sensors compared. Phys. Stat. Sol. (A).

[3] Grilj V., Skukan N., Jakšić M., Pomorski M., Kada W., Kamiya T., Ohshima T. (2016). The evaluation of radiation damage parameter for CVD diamond. Nucl. Instrum. Methods Phys. Res. Sect. Beam Interact. Mater. Atoms.

[4] Skukan N., Sudić I., Pomorski M., Kada W., Jakšić M. (2019). Enhanced radiation hardness and signal recovery in thin diamond detectors. AIP Adv..

[5] Tsubota M., Kaneko J.H., Miyazaki D., Shimaoka T., Ueno K., Tadokoro T., Chayahara A., Watanabe H., Kato Y., Shikata S. (2015). High-temperature characteristics of charge collection efficiency using single CVD diamond detectors. Nucl. Instrum. Methods Phys. Res. Sect. Accel. Spectrometers Detect. Assoc. Equip..

[6] Steinegger P., Dressler R., Eichler R., Piguet D., Streuli S., Türler A. (2017). Diamond detectors for high-temperature transactinide chemistry experiments. Nucl. Instrum. Methods Phys. Res. Sect. Accel. Spectrometers Detect. Assoc. Equip..

[7] Angelone M., Cesaroni S., Loreti S., Pagano G., Pillon M. (2019). High temperature response of a single crystal CVD diamond detector operated in current mode. Nucl. Instrum. Methods Phys. Res. Sect. Accel. Spectrometers Detect. Assoc. Equip..

[8] Umezawa H. (2018). Recent advances in diamond power semiconductor devices. Mater. Sci. Semicond. Process..

[9] Optris PI 640 The Smallest Measuring VGA Thermal Imager Worldwide. https://www.optris.global/thermal-imager-optris-pi-640.

[10] Cosic D., Bogovac M., Jakšić M. (2019). Data acquisition and control system for an evolving nuclear microprobe. Nucl. Instrum. Methods Phys. Res. Sect. Beam Interact. Mater. Atoms.

[11] Jakšić M., Bogdanović Radović I., Bogovac M., Desnica V., Fazinić S., Karlušić M., Medunić Z., Muto H., Pastuović Ž., Siketić Z. (2007). New capabilities of the Zagreb ion microbeam system. Nucl. Instrum. Methods Phys. Res. Sect. Beam Interact. Mater. Atoms.

[12] Breese M.B.H., Vittone E., Vizkelethy G., Sellin P.J. (2007). A review of ion beam induced charge microscopy. Nucl. Instrum. Methods Phys. Res. Sect. Beam Interact. Mater. Atoms.

[13] McMath T.A., Martini M. (1970). The effect of charge trapping on the spectrometer performance of p-i-n semiconductor detectors. Nucl. Instrum. Methods.

[14] Marinelli M., Milani E., Paoletti A., Tucciarone A., Verona-Rinati G., Angelone M., Pillon M. (2001). Trapping and detrapping effects in high-quality chemical-vapor-deposition diamond films: Pulse shape analysis of diamond particle detectors. Phys. Rev. B.

[15] Vittone E. (2004). Theory of ion beam induced charge measurement in semiconductor devices based on the Gunn’s theorem. Nucl. Instrum. Methods Phys. Res. Sect. Beam Interact. Mater. Atoms.

[16] Sato S., Makino T., Ohshima T., Kamiya T., Kada W., Hanaizumi O., Grilj V., Skukan N., Pomorski M., Vizkelethy G. (2017). Transient current induced in thin film diamonds by swift heavy ions. Diam. Relat. Mater..

[17] Kada W., Iwamoto N., Satoh T., Onoda S., Grilj V., Skukan N., Koka M., Ohshima T., Jakšić M., Kamiya T. (2014). Continuous observation of polarization effects in thin SC-CVD diamond detector designed for heavy ion microbeam measurement. Nucl. Instrum. Methods Phys. Res. Sect. Beam Interact. Mater. Atoms.

[18] Naaranoja T., Golovleva M., Martikainen L., Berretti M., Österberg K. (2019). Space charge polarization in irradiated single crystal CVD diamond. Diam. Relat. Mater..

[19] Pan L.S., Kania D.R. (1995). Diamond: Electronic Properties and Applications.

[20] Ziegler J.F., Ziegler M.D., Biersack J.P. (2010). SRIM—The stopping and range of ions in matter. Nucl. Instrum. Methods Phys. Res. Sect. Beam Interact. Mater. Atoms.

[21] Huhtinen M. (2002). Simulation of non-ionising energy loss and defect formation in silicon. Nucl. Instrum. Methods Phys. Res. Sect. Accel. Spectrometers Detect. Assoc. Equip..

[22] Pintilie I., Lindstroem G., Junkes A., Fretwurst E. (2009). Radiation-induced point- and cluster-related defects with strong impact on damage properties of silicon detectors. Nucl. Instrum. Methods Phys. Res. Sect. Accel. Spectrometers Detect. Assoc. Equip..

[23] Bachmair F. (2016). Diamond sensors for future high energy experiments. Nucl. Instrum. Methods Phys. Res. Sect. Accel. Spectrometers Detect. Assoc. Equip..

[24] Skukan N., Grilj V., Sudić I., Pomorski M., Kada W., Makino T., Kambayashi Y., Andoh Y., Onoda S., Sato S. (2016). Charge multiplication effect in thin diamond films. Appl. Phys. Lett..

[25] Nava F., Canali C., Artuso M., Gatti E., Manfredi P.F., Kozlov S.F. (1979). Transport Properties of Natural Diamond Used as Nuclear Particle Detector for a Wide Temperatue Range. IEEE Trans. Nucl. Sci..

[26] Tanimura Y., Kaneko J., Katagiri M., Yujiro I., Nishitani T., Takeuchi H., Iida T. (2000). High-temperature operation of a radiation detector made of a type IIa diamond single crystal synthesized by a HP/HT method. Nucl. Instrum. Methods Phys. Res. Sect. Accel. Spectrometers Detect. Assoc. Equip..

[27] Kumar A., Kumar A., Topkar A., Das D. (2017). Prototyping and performance study of a single crystal diamond detector for operation at high temperatures. Nucl. Instrum. Methods Phys. Res. Sect. Accel. Spectrometers Detect. Assoc. Equip..

[28] Gabrysch M., Majdi S., Twitchen D.J., Isberg J. (2011). Electron and hole drift velocity in chemical vapor deposition diamond. J. Appl. Phys..

[29] Kramberger G., Cindro V., Mandić I., Mikuž M., Zavrtanik M. (2002). Effective trapping time of electrons and holes in different silicon materials irradiated with neutrons, protons and pions. Nucl. Instrum. Methods Phys. Res. Sect. Accel. Spectrometers Detect. Assoc. Equip..

[30] Bruzzi M., Menichelli D., Sciortino S., Lombardi L. (2002). Deep levels and trapping mechanisms in chemical vapor deposited diamond. J. Appl. Phys..

[31] Iakoubovskii K., Kiflawi I., Johnston K., Collins A., Davies G., Stesmans A. (2003). Annealing of vacancies and interstitials in diamond. Phys. Condens. Matter..

[32] Ueda K., Kawamoto K., Soumiya T., Asano H. (2013). High-temperature characteristics of Ag and Ni/diamond Schottky diodes. Diam. Relat. Mater..

